# From Passive to Active—Improving the Healthy Self-Help Behavior of Older Adults Through Community Health Association: Mixed Methods Study

**DOI:** 10.2196/81062

**Published:** 2025-11-25

**Authors:** Xinxin Wang, Chengrui Zhang, Yue Qi, Ying Xing, Zhu Zhu, Lan-Shu Zhou, Wei Luan

**Affiliations:** 1 Shuguang Hospital Affiliated to Shanghai University of Traditional Chinese Medicine, 528 Zhangheng Road, Pudong New Area, Shanghai, China, 86 021-20256016; 2 School of Nursing, Shanghai Jiao Tong University, Shanghai, China; 3 School of Health Management, Harbin Medical University, Harbin, China; 4 School of Nursing, Naval Medical University, Shanghai, China

**Keywords:** health association, healthy behavior, health education, mixed methods study, older adults

## Abstract

**Background:**

While China’s aging population and strained health care resources heighten the need for effective health promotion, traditional community health education faces barriers such as passive participation among older adults, short-term behavioral changes, and limited sustainability.

**Objective:**

This study aims to develop and examine the impact of an innovative community healthy self-help education model for older adults on healthy behavior and active health awareness among older people.

**Methods:**

A mixed methods study was conducted to enroll older participants, including a 12-month pre-post self-controlled trial in 5 communities in Shanghai, China. Health behaviors, autonomy, and eHealth literacy were assessed at baseline, 6 months, and 12 months using standardized scales (measuring health-promoting lifestyle, self-rated abilities for health practices, healthy self-management behaviors, participation/autonomy, and eHealth literacy). Comparisons of scale scores at each time point were analyzed using repeated measures ANOVA. Semistructured interviews were conducted after the intervention, focusing on the dimensions of willingness to manage health, behavioral transformation, social role change, and attend experiences. The themes were extracted through thematic analysis. Qualitative data served to interpret and enrich quantitative findings.

**Results:**

A total of 80 community-based older people were included in our study, with a mean age of 68.9 (SD 2.2) years. Intervention participants significantly improved in healthy self-help behavior (*F*_3,237_=25.43, *P*<.001). The total mean score improved from 85.90 (SD 22.74) baseline to 107.46 (SD 16.09) 12 months post intervention. Sustained enhancements occurred in health promotion lifestyle (*F*_3,237_=76.41, *P*<.001), health practices ability (*F*_3,237_=31.82, *P*<.001), participation and autonomy (*F*_3,237_=5.11, *P*=.004), and eHealth literacy (*F*_3,237_=26.75, *P*=.002). At the end of the intervention, 11 participants attended semistructured interviews. After the intervention, older people demonstrated stronger willingness and proactive behavior in health management, with increased health knowledge and social engagement. Compared to self-directed activities, health care professional–led education was perceived as more authoritative, whereas peer-organized activities were more interactive and flexible.

**Conclusions:**

The community health association education model based on a community healthy self-help education model for older adults significantly enhanced older adults’ healthy self-management behavior, active health awareness, and eHealth literacy. Integrating professional support with peer empowerment addressed core limitations of traditional models: low engagement and unsustainable behavioral change. This community-embedded approach provides a scalable solution for sustainable health promotion, with significant policy implications for alleviating health care system pressures and advancing active aging.

## Introduction

China is in a period of accelerated aging, and the proportion of older people continues to rise, posing a serious challenge to the public health and medical service system [1,2]. The “Healthy China 2030” program proposes transitioning from “passive medical care” to “active health,” emphasizing that older people should become active managers of their well-being [3]. Studies have shown that self-determination and self-management of older people can improve health [4,5]. Healthy self-help behavior can improve quality of life, delay chronic diseases, reduce health care dependency [6-9], and effectively complement the gap in social health services [10-12].

There are limitations in the current community health promotion model: first, the health education model is passively dependent on health care worker domination, and there is a lack of cultivation of endogenous motivation [6,13-15]. Second, sustainability is insufficient, often due to short-term funding cycles or a lack of institutional support for long-term program maintenance [16,17]. Third, the poor interactivity of the educational format restricts the formation of social networks among older people [18,19]. Older people’s intrinsic motivation to actively seek health information and use resources is insufficient, and their ability to acquire and apply health knowledge is limited, which hinders their fundamental transformation to the role of “active health.”

The path to breakthrough lies in designing models that facilitate the transformation of older people into active leaders. As a form of organization relying on community resources, community health associations have the potential to enhance social participation, promote peer support, and empower individuals [20-22]. Our study aims to examine the effectiveness of our community healthy self-help education model for older adults (CHSE-O) in facilitating the transition from passive reception to active engagement in health self-management among older adults. Specifically, we seek to (1) evaluate the impact of the CHSE-O model on improving health-related knowledge, self-management capacity, social participation, and sustainable healthy behaviors and (2) explore how social participation and peer support within community health associations foster motivation and behavioral change. Through this mixed methods investigation, we intend to provide actionable insights for constructing a feasible and scalable community-based health promotion system tailored to the needs of an aging population.

## Methods

### Educational Program Design

#### Conceptual Framework

Our study team has developed an operational definition and classification of the concept of “healthy self-help” based on the traceability and evolutionary analysis of the concept [4,5]. Based on self-care and self-management theory, the concept emphasizes that in the whole health care process, health care professionals can assist older people in completing the transformation of their roles and behaviors from passive to active and from vulnerable to dominant, along with the emergence and development of healthy self-help behavior of patients. The core of the concept lies in the willingness of individuals to take the initiative to maintain their health and the spontaneous formation of healthy self-help behaviors, including the active acquisition, communication, and application of health information and medical resources, in order to achieve the goals of health maintenance, disease treatment, and rehabilitation [4,5].

**Figure 1.**
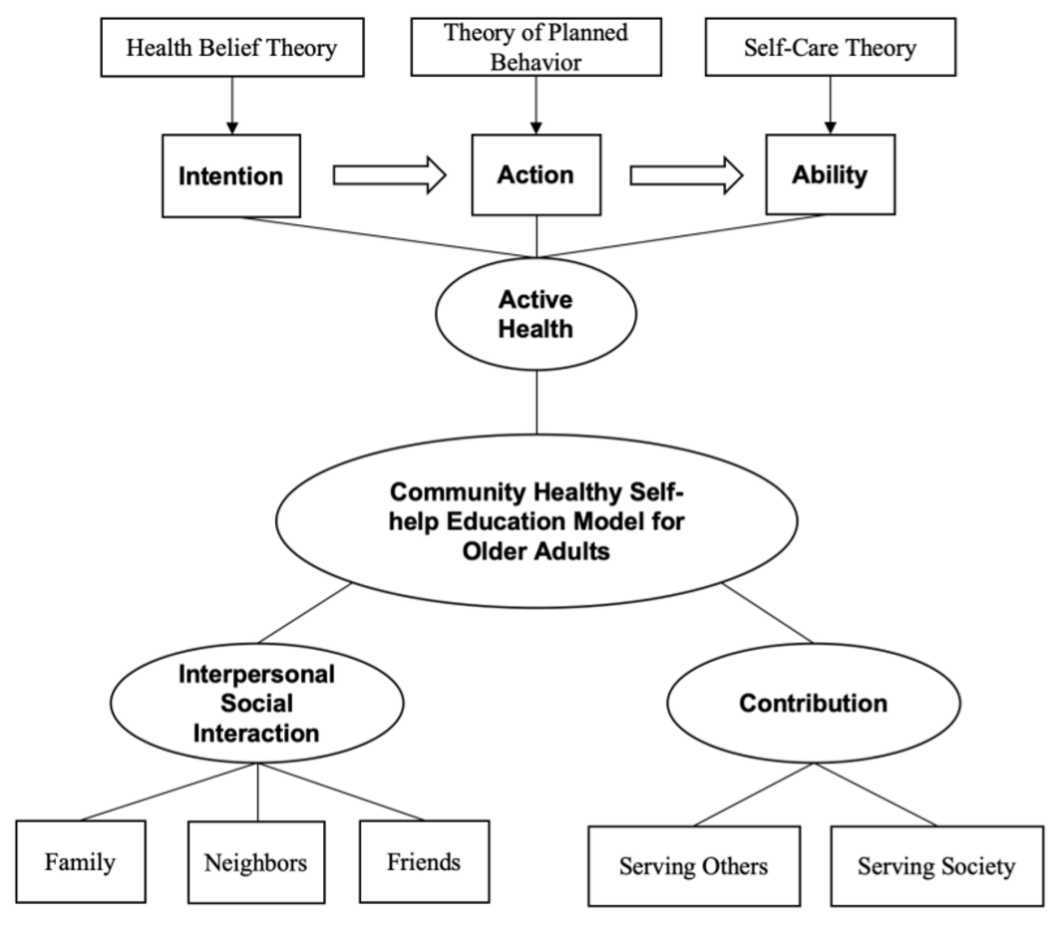
Conceptual framework for a community healthy self-help education model for older adults (CHSE-O).

Our study framework contains 3 key dimensions (Figure 1). The active health dimension stimulates older people’s willingness to take charge of their health, promotes the transformation of knowledge into action, and ultimately enhances their independent management ability to realize the transformation from passive acceptance to active control. The interpersonal social interaction dimension focuses on building a mutual support network, promoting emotional connection and sharing health knowledge among older people, and alleviating social isolation. The social contribution dimension aims to stimulate the value of the “worthiness of the older people,” guide them to change from health knowledge recipients to community health promoters, and strengthen their social identity by serving others, thus forming a virtuous circle.

#### Constructing a Community Health Association Education Model for Older Adults

**Figure 2.**
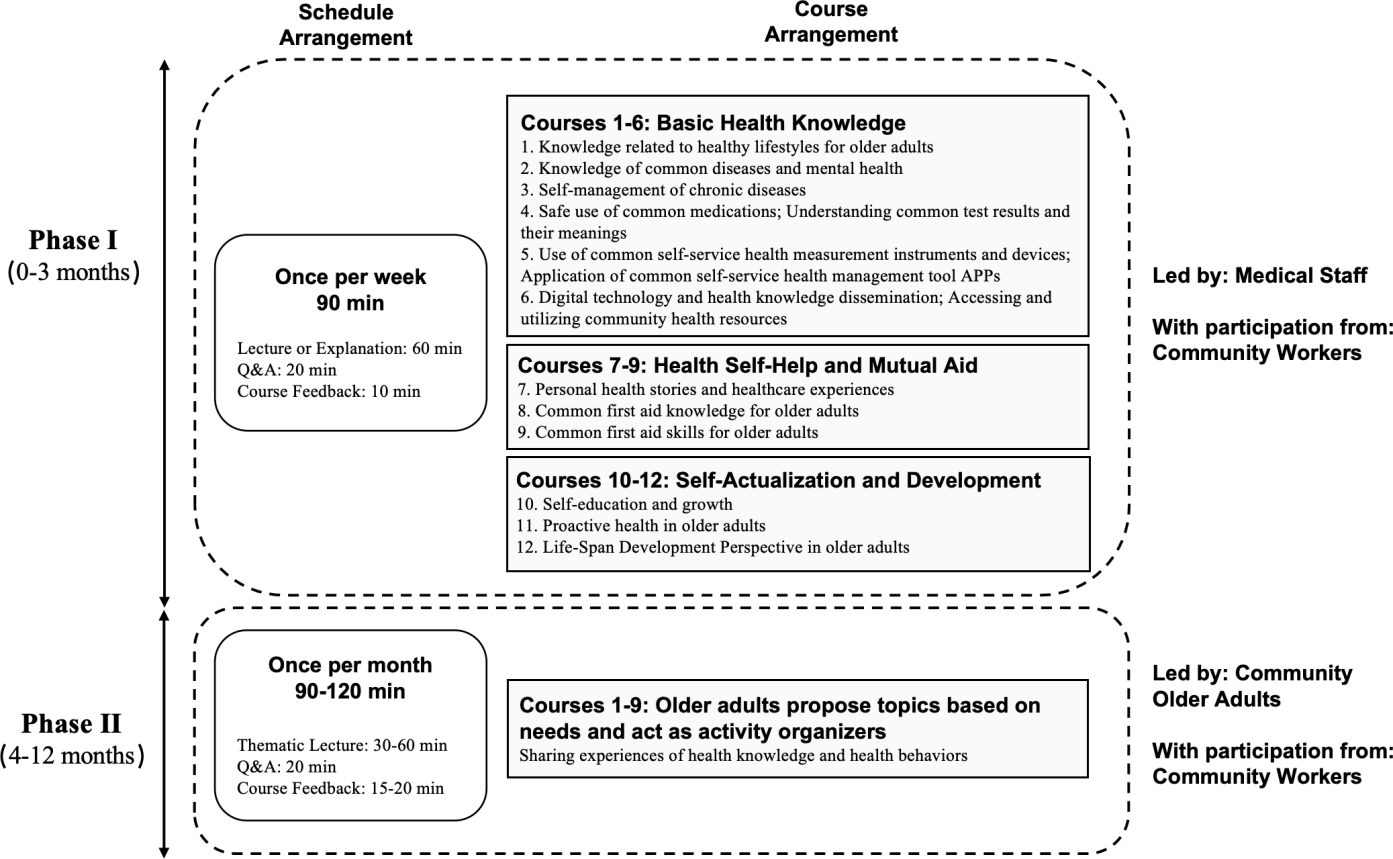
Community health association education model based on a community healthy self-help education model for older adults (CHSE-O).

**Figure 3.**
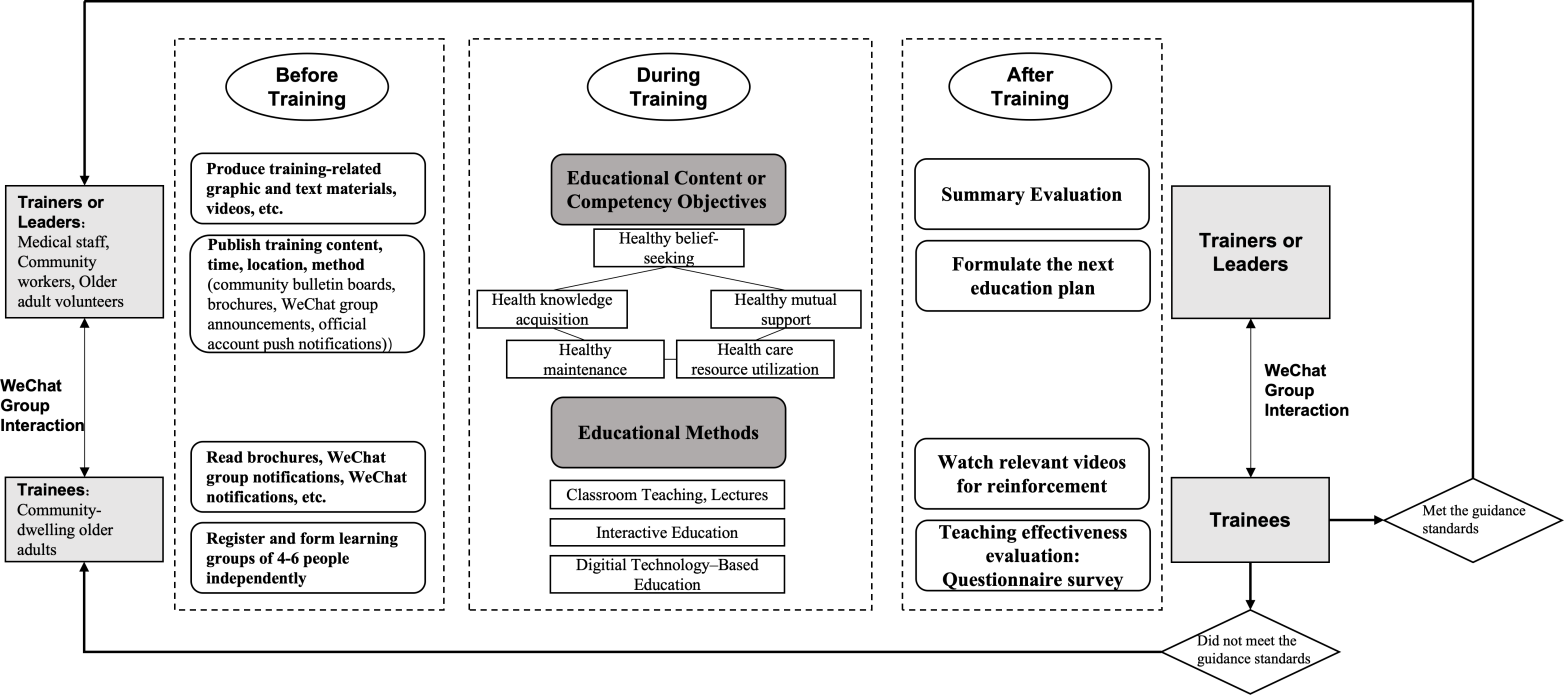
The implementation program of a community health association education model based on a community healthy self-help education model for older adults (CHSE-O).

Through sorting out specific cases and experiences of health education practice research for older adults, the community health association education model based on CHSE-O (Figure 2) and a specific implementation program (Figure 3) were sorted out based on the conceptual framework, combining expert opinions and the physiological and psychological characteristics of older people.

#### Expert Group Meetings

Our study chose an expert group meeting to discuss the scientific validity and feasibility of the educational program. The selection criteria for the experts were (1) more than 10 years of work experience in the field of medical and nursing practice, more than 5 years of work experience for community workers, (2) physicians need to have a technical title of associate senior or above, nursing staff need to have a technical title of intermediate or above, no title is required for community workers, and (3) willingness to participate in the study. The corresponding author (WL) recruited experts through her personal research network and applied a snowball sampling method to expand the participant pool further. A total of 18 experts participated in the expert group meeting, including 2 community general practitioners, 8 senior nurses, 6 intermediate nurses, and 2 community workers. The outline of the meeting included the following issues: (1) exploring the construction of a conceptual framework for a community health education model for older people, (2) assessing the feasibility and innovativeness of the program in theory and practice, and (3) making specific recommendations to optimize the program. The flow and main contents of the expert group meeting are shown in Multimedia Appendix 1.

#### Participants and Data Collection: Recruitment and Administration

This 12-month intervention study was conducted using a pre-post self-controlled design from June 2023 to June 2024 in Shanghai, China. Data collection was conducted face-to-face at months 0, 3, 6, and 12 of the intervention. Individuals who showed their willingness to participate in the study were provided with verbal and written information about the objectives and characteristics of the study.

Our study selected 5 communities (Zong Tong, Chang Feng Fang, Zi Jing Yuan, Hua Jing, and Yu Qiao) in Shanghai, China, to conduct a study on applying a community health association education program for older people. Patient recruitment comprises 2 phases: initial screening and enrollment assessment. In the initial stage, community workers collaborating with our research team will provide a brief introduction to the project for older people within the community. In the eligibility assessment phase, older people preliminarily screened by the research team and meeting the inclusion criteria will be invited to participate in the study. The inclusion criteria for participants were as follows: (1) signing an informed consent form and willing to participate in the study; (2) aged 60 years or older and having lived in the community for more than 6 months; and (3) possessing complete communication language skills, including hearing comprehension, oral expression, reading comprehension, and written expression. Exclusion criteria were (1) a diagnosis of a mental disorder (eg, schizophrenia, delusions) or cognitive disorder (eg, Alzheimer disease, vascular cognitive impairment) and (2) a diagnosis of a major or advanced terminal illness, such as malignant neoplasm or end-stage renal disease.

The G*Power software (version 3.1.7; G*Power, Germany) was used, taking a test efficacy of 0.8, a significance level of *α*=.05, and an effect size estimate of 0.5 based on previous studies in the literature [4]. Our study had a sample size of at least 64 cases after considering a 20% loss to follow-up rate. During the first stage, the research team contacted 215 older adults, of whom 22 expressed no interest and 15 indicated they lacked the time to participate. After eligibility screening, 13 individuals were ineligible for participation. A total of 165 participants were enrolled at baseline, with 162 retained at 3-month follow-up, 112 at 6-month follow-up, and 80 completing all study phases.

### Ethical Considerations

The study was approved by the Ethics Committee of Renji Hospital, affiliated with Shanghai Jiao Tong University School of Medicine (approval number RA-2021‐465). Participants understood the study’s purpose, process, risks, and benefits and voluntarily agreed to participate. Information and informed consent forms were provided, and participants were required to sign both documents before the study began. The survey adhered to anonymity and confidentiality, and responses were collected anonymously. Small gifts worth 20 RMB (equivalent to US $3; such as tableware, traditional Chinese medicine health care products) were provided to participants as compensation.

### Evaluation of the Effectiveness of Educational Program

#### Integration of Quantitative and Qualitative Data

Our study utilized an explanatory sequential mixed methods design, using the quantitative questionnaire survey first and semistructured interviews based on the quantitative findings [23]. Qualitative data served to interpret and enrich quantitative findings, with both parts analyzed separately prior to integration. Through merging, quantitative discoveries were compared and integrated with themes emerging from qualitative analysis, ultimately yielding a comprehensive interpretation [24].

#### Quantitative Methods

##### Research Tools

###### General Information Questionnaire

It was designed by the researcher, including gender, age, educational level, religious beliefs, marital status, residential situation, household monthly income, primary source of income, medical payment methods, signed long-term care insurance or not, self-assessed health status, and number of chronic diseases.

###### Health Promotion Lifestyle Scale

Our study used the Health Promotion Lifestyle Scale developed by Walker et al [25]. A total of 52 entries were divided into 6 dimensions: health responsibility, nutrition, physical activity, interpersonal relationships, stress management, and spiritual growth. The total score ranges from 52 to 208, and the higher the score, the higher the level of healthy lifestyle. The scale demonstrated high internal consistency (*α*=.910).

###### Self-Rated Abilities for Health Practices Scale

Becker developed the scale comprising 28 entries, including 4 subscales: nutrition, psychological well-being, exercises, and responsible health practices [26]. According to the self-assessment of the individual’s mastery of health behaviors, “almost no certainty” scored 0, “a little certainty” scored 1, “moderate certainty” scored 2, “greater certainty” scored 3, “absolute certainty” scored 4, and the higher the score, the higher the self-efficacy of the individual in health behaviors. The scale demonstrated high internal consistency (*α*=.958).

###### Healthy Self-Help Behavior Questionnaire

The Healthy Self-Help Behavior Questionnaire was developed by Lin [27]. It was divided into 5 dimensions, comprised 26 entries, and scored on a 5-point Likert scale. The questionnaire was based on 5 dimensions: healthy belief-seeking, health knowledge acquisition, healthy mutual support, healthy maintenance, and health care resource utilization. Higher scores represent higher levels of healthy self-help behavior. The scale demonstrated high internal consistency (*α*=.954).

###### Impact on Participation and Autonomy

The original scale was developed by Cardol et al [28] to measure patients’ self-perceived level of social participation. Our study used the Chinese version of the questionnaire introduced by Li Hong and revised in Chinese [29]. The revised questionnaire includes 4 dimensions, namely indoor autonomous participation, family role autonomous participation, outdoor autonomous participation, and social life autonomous participation, with a total of 25 entries. Respondents were asked to choose from “fully compliant” to “not at all compliant” according to their situation, with scores ranging from 0 to 4. The higher the score, the poorer the level of social participation. Standardized score = (average of each dimension/sum of the full score of each question)×100%. The scale demonstrated high internal consistency (*α*=.940).

###### The eHealth Literacy Scale

The eHealth Literacy Scale (eHEALS) was developed by Norman et al [30] and consists of 8 questions assessing the ability to apply online health information and services, judgment, and decision-making. The scale is based on a 5-point Likert scale ranging from 8 to 40, with higher scores indicating higher eHealth literacy. The scale demonstrated high internal consistency (*α*=.982).

### Data Analysis

Excel 2019 (Microsoft Corporation) was used for raw data entry, and SPSS 25.0 (IBM Corporation) statistical software was used for statistical analysis. Sociodemographic information was expressed as the number of cases and percentages. Measurement information was statistically described using means and standard deviations, and for repeated measures, repeated measures ANOVA was used for statistical inference. Differences were considered statistically significant at *P*<.05. Our study observed no significant differences in intervention effects across participants’ demographic characteristics (all *P*>.05). Therefore, the repeated measures model did not include these variables as covariates.

### Qualitative Methods

#### Data Collection

After the intervention, semistructured interviews were conducted using a convenience sampling method of 5 from each community, with 2-3 older people participating throughout. Interviews were conducted by uniformly trained researchers (XW and CZ) in quiet, private spaces within community older people activity centers. After obtaining informed consent from the interviewees, all interviews were fully recorded and transcribed verbatim into text materials for analysis. All potential interview participants were informed during the quantitative survey phase in the early stages of the project that our study included an interview component, and they were made aware of the primary purpose and general content of the interviews. Prior to the commencement of the actual interview, the researcher will once again provide the interviewee with a detailed explanation of the research objectives, privacy protection measures, and data usage rights and obtain a signed written informed consent form.

The interview outlines focused on changes in older people’s (1) willingness to manage their health, (2) health behavior formation, (3) health-related competence enhancement, (4) changes in attitudes toward health and active aging, (5) comparisons of experiences with the health education model, and (6) opinions and recommendations after receiving the community health education model. Each interview lasted 20-40 minutes and was audio-recorded for subsequent verbatim transcription and translation. The interview portion of the study was discontinued after all authors agreed on thematic saturation.

#### Data Analysis

Our analysis of the interview texts followed a thematic analysis process. All interview texts were imported into and managed using the NVivo 12 qualitative analysis software (version 12; QSR International). Multiple researchers conducted coding collaboratively to ensure analytical rigor and minimize individual subjective bias. Research team members XW, CZ, and YQ each independently read through the entire interview text prior to analysis, and initial themes were identified through open coding combined with deductive and inductive strategies. The assessment of thematic saturation runs throughout the iterative data collection and analysis process. After completing approximately half of the interviews, we conducted preliminary analysis concurrently. Subsequently, after every 2‐3 new interviews were completed, the research team convened coding consensus meetings to review whether the new data introduced novel themes or dimensions to the coding framework. When consecutive new data no longer yield fresh insights and existing themes have been sufficiently developed and enriched, thematic saturation is assessed as achieved. Our study confirmed saturation after completing the 11th interview and ceased recruiting additional participants. Subsequently, XW developed the preliminary coding framework through thematic comparison and multiple discussions among several coders. The coding team held multiple consensus meetings where coders compared their independently coded results and discussed discrepancies until agreement was reached. This process was conducted under a senior researcher’s (WL) guidance. After multiple rounds of discussion and refinement, a clearly structured and well-defined coding list was ultimately established.

## Results

### Quantitative Results

#### Sociodemographic Characteristics of Participants

A total of 80 community-based older people were included in our study, of whom 15 (18.8%) were male and 65 (81.3%) were female; their ages ranged from 60 to 99 years old, with a mean age of 68.9 (SD 2.2) years. About 60% (48/80) of the participants had an education level of junior high school or below, 83.8% (67/80) had no religious beliefs, 82.5% (66/80) were married, and 52.5% (42/80) were living with their spouses. Monthly household income was mainly less than RMB 5000 (equivalent to US $700; 56.3%), 93.8% (75/80) had a pension as their primary source of income, and 76.3% (61/80) paid for their medical expenses through municipal health insurance. About 91.3% (73/80) of the participants had not signed up for long-term care insurance. Most participants rated their health as “Average” (45.0%), and 73.8% (59/80) had at least 1 chronic disease (Multimedia Appendix 2).

##### Changes in Scale Scores at Each Stage of the Intervention

###### Health Promotion Lifestyle Scale

The dimensions of health responsibility, nutrition, physical activity, interpersonal relationships, stress management, and spiritual growth were significantly higher after the intervention (*P*<.001). The total score improved from 94.41 (SD 18.58) at baseline to 123.19 (SD 18.90) at 12 months post-intervention with a statistically significant difference (*F*_3,237_=76.41, *P*<.001), as shown in Table 1.

**Table 1.** Comparison of scale scores by time point.

Dimensions	Baseline, mean (SD)	3-month, mean (SD)	6-month, mean (SD)	12-month, mean (SD)	*F* test (*df*)	*P* value
HPLP II^a^						
Health responsibility	23.08 (5.64)	30.16 (5.17)	31.89 (6.38)	32.84 (6.18)	44.34 (3, 237)	<.001
Nutrition	16.03 (3.17)	19.34 (3.47)	18.89 (2.92)	19.53 (3.29)	20.32 (3, 237)	<.001
Physical activity	15.96 (4.78)	20.44 (3.92)	22.46 (5.07)	21.9 (4.89)	30.62 (3, 237)	<.001
Interpersonal relationships	13.56 (3.69)	14.56 (2.70)	16.41 (2.44)	16.60 (2.81)	17.77 (3, 237)	<.001
Stress management	12.82 (3.46)	14.39 (2.56)	16.24 (2.67)	16.11 (2.80)	23.61 (3, 237)	<.001
Spiritual growth	12.86 (3.74)	14.63 (2.65)	16.06 (2.69)	16.21 (2.67)	20.94 (3, 237)	<.001
Total score	94.41 (18.58)	113.51 (15.66)	121.95 (17.18)	123.19 (18.90)	76.41 (3, 237)	<.001
SRAHP^b^						
Nutrition	21.41 (5.73)	25.94 (4.56)	27.28 (4.58)	27.99 (4.39)	29.54 (3, 237)	<.001
Psychological well-being	21.19 (6.91)	25.64 (4.84)	27.25 (4.95)	27.76 (4.42)	24.18 (3, 237)	<.001
Exercises	19.34 (6.89)	25.10 (4.99)	26.64 (5.45)	27.22 (4.57)	33.87 (3, 237)	<.001
Responsible health practices	21.14 (6.88)	25.38 (4.69)	26.85 (4.85)	27.39 (4.56)	22.44 (3, 237)	<.001
Total score	83.07 (23.94)	102.05 (18.23)	108.01 (18.63)	110.36 (16.93)	31.82 (3, 237)	<.001
Healthy Self-help Behavior						
Healthy belief-seeking	9.70 (2.73)	10.95 (2.11)	11.83 (1.98)	12.16 (1.95)	19.89 (3, 237)	<.001
Health knowledge acquisition	9.47 (2.85)	10.99 (2.11)	11.76 (1.85)	12.16 (2.08)	21.26 (3, 237)	<.001
Healthy mutual support	9.54 (2.88)	10.74 (2.16)	11.66 (2.11)	12.06 (2.15)	18.30 (3, 237)	<.001
Healthy maintenance	10.06 (2.85)	10.80 (2.16)	11.86 (2.13)	12.21 (2.13)	14.21 (3, 237)	<.001
Health care resource utilization	47.12 (12.87)	52.95 (8.75)	58.31 (8.05)	58.86 (8.87)	25.84 (3, 237)	<.001
Total score	85.90 (22.74)	96.43 (16.22)	105.43 (14.28)	107.46 (16.09)	25.43 (3, 237)	<.001
IPA-I^c^						
Indoor autonomous participation	10.39 (5.89)	11.51 (6.41)	10.03 (5.19)	8.98 (4.88)	2.81 (3, 237)	.04
Family role autonomous participation	12.19 (5.88)	11.81 (6.14)	10.70 (5.18)	9.35 (4.41)	4.55 (3, 237)	.004
Outdoor autonomous participation	6.65 (3.28)	6.60 (3.42)	5.56 (2.77)	5.40 (2.75)	3.86 (3, 237)	.01
Social life autonomous participation	11.69 (5.19)	12.71 (6.09)	10.06 (4.38)	9.39 (4.43)	7.58 (3, 237)	<.001
Total score	40.91 (17.64)	42.64 (21.05)	36.35 (16.12)	33.11 (14.74)	5.11 (3, 237)	.004
eHEALS^d^	25.06 (6.84)	28.78 (6.28)	32.29 (4.98)	32.31 (5.14)	26.75 (3, 237)	.002

a HPLP II: Health Promotion Lifestyle Scale.

b SRAHP: Self-rated Abilities for Health Practices Scale.

c IPA-I: Impact on Participation and Autonomy.

d eHEALS: eHealth Literacy Scale.

###### Self-Rated Abilities for Health Practices Scale

The dimensions of nutrition, psychological well-being, exercises, and responsible health practices were significantly higher after the intervention (*P*<.001). The total score improved from 83.07 (SD 23.94) at baseline to 10.36 (SD 16.93) at 12 months post-intervention with a statistically significant difference (*F_3,237_*=31.82, *P*<.001), as shown in Table 1.

###### Healthy Self-Help Behavior Questionnaire

The dimensions of healthy belief-seeking, health knowledge acquisition, healthy mutual support, healthy maintenance, and health care resource utilization were significantly higher after the intervention (*P*<.001). The total score improved from 85.90 (SD 22.74) at baseline to 107.46 (SD 16.09) at 12 months post-intervention with a statistically significant difference (*F_3,237_*=25.43, *P*<.001), as shown in Table 1.

###### Impact on Participation and Autonomy

Lower scores on the IPA-I scale indicate higher autonomy of the participants. The study’s results showed that scores on all dimensions decreased after the intervention. The total score on the IPA-I scale decreased from 40.91 (SD 17.641) at baseline to 33.11 (SD 14.74) 12 months after the intervention (*F_3,237_*=5.11, *P*=.004), indicating a significant increase in the overall autonomy of the participants, as shown in Table 1.

###### eHealth Literacy Scale

Participants’ eHEALS total score significantly improved from 25.06 (SD 6.84) at baseline to 32.31 (SD 5.14) at 12 months post-intervention, with a statistically significant difference (*F_3,237_*=26.75, *P*=.002), as shown in Table 1.

###### Participant Satisfaction

The overall satisfaction score (1‐5 points: very dissatisfied to very satisfied) of participants with the health education program was 4.55 (SD 0.59), with high satisfaction with the content, mode, education implementer, effect, and environment, and all participants were willing to recommend others and participate in similar education activities again, as shown in Table 2.

**Table 2.** Participant satisfaction scores.

Items	Mean (SD)		Min	Max
How satisfied are you overall with the health education program we provide?	4.55 (0.59)		3	5
How satisfied are you with the content of the education?	4.53 (0.67)		2	5
How satisfied are you with the way the education was delivered?	4.57 (0.59)		3	5
How satisfied are you with the educators?	4.61 (0.58)		3	5
How satisfied are you with the effectiveness of the program?	4.56 (0.67)		2	5
How satisfied are you with the educational environment and facilities?	4.60 (0.67)		3	5

### Qualitative Findings

#### General Information of the Participants

At the end of the intervention, 11 participants were selected to be interviewed, and the general information about the interviewees is shown in Multimedia Appendix 3.

#### Thematic Findings

##### Increased Willingness to Manage Health

In this study, participants generally showed a strong willingness to be active in health management, believing that health is central to the quality of life in later life (eg, A8: “Health is the most important thing right now”), and several participants clearly expressed this willingness. Health crises became an important driver, for example, A8 (postcardiac stenting) and A10 (postthyroid surgery) placed more emphasis on health management due to experiencing significant health problems; trust in health care organizations also facilitated active participation, for example, A9, A10, and A1 actively participated in relevant activities due to trust in the hospitals; and family roles also played a role, with A3 and A8 mentioning that “Children are busy at work and need self-management,” indicating that they want to reduce the burden on the family through self-management.

##### Changes in Health Behaviors

In terms of changes in health behaviors, participants significantly improved their health by adjusting their dietary control and exercise habits, with A5 mentioning that she started practicing moxibustion and acupressure after attending a Chinese medicine course, and A10 adjusting her lifestyle habits after discovering a thyroid nodule at a health seminar and having it operated on promptly. In addition, participants actively expanded their sources of health information, with A3 obtaining information through newspapers, A7 using Baidu and public websites, and A9 following TV health programs and short videos. It is worth noting that A7 emphasized that she “pays attention to the sources and distinguishes between advertisements and popular science,” reflecting the importance that participants place on their ability to filter information.

##### Health Knowledge and Skills Enhancement

Regarding health knowledge and skill enhancement, participants significantly enhanced their health literacy through traditional Chinese medicine health care practices and chronic disease management. At the end of the education, participants began to try moxibustion, Baduanjin, and health exercises (A6, A7, and A9), while A7 improved his confidence in traditional Chinese medicine health care due to his participation. Regarding chronic disease management, A8 took medication regularly after surgery, A10 self-monitored his thyroid status after surgery, and A3 helped adjust the diet of the orphaned older adults by pairing them up. A3 mentioned that “when the paired older people resisted the knowledge of the disease, they learned to communicate with each other gently to alleviate their depression,” and A10 learned the importance of medical check-ups through the society’s activities, timely detection of health hazards, and intervention. A10 learned the importance of medical check-ups through the association’s activities to detect health risks and intervene in time. In addition, the participants also played an active role in health popularization, with A3 promoting health knowledge through twinning, A6 actively sharing knowledge of Chinese medicine, and A8 organizing discussions on health knowledge. At the family level, A5 and A7 shared what they had learned with their children, effectively raising the family’s health awareness.

##### Social Skills and Community Interaction

Community integration was achieved through the participation of multiple roles, such as old partner pairing, community volunteers, activity promoters, WeChat group leaders, and so forth, and the social circle of the participants was significantly expanded as a result. A8 got to know more neighbors through participation in community activities, and the frequency of sharing health knowledge was significantly increased in her daily exchanges; in the process of helping the older adults who lived alone, A3 not only built up deeply trusting relationships but also further participated in the life care of the other party to form a closer social bond. While helping older people living alone, A3 established a deep relationship of trust and participated in each other’s life care, forming a closer social connection. In terms of preferred forms of interaction, A3 and A7 showed higher recognition of activities led by health care professionals, believing that they were professional and well prepared and especially trusting in the authority of Shuguang Hospital; A6, A2, and A9 preferred self-organized activities, enjoying the relaxing atmosphere of calling on friends and family members to share their experiences, with A6 mentioning in particular that “communication is more comfortable with peers,” and A6 said, “it is more comfortable with peers.” A6 particularly mentioned that “people of the same age are more comfortable communicating with each other,” and that this form of communication made them feel relaxed and intimate.

##### Comparison of Medical Staff-Led and Older People’s Independent Participation in Health Education Experience

Significant differences were observed in the form and content of health education activities led by medical staff compared to those organized independently by older adults. Activities led by medical personnel were characterized by systematic curricula and high professionalism, with participants A7 and A3 emphasizing their “high credibility” and “strong knowledge authority,” respectively. In contrast, older people’s autonomous activities were noted for their flexibility and alignment with practical needs, as highlighted by participants A2 and A6, who described them as “more relevant to daily life.” In terms of interactivity, medical staff-led activities primarily followed a 1-way teaching approach, whereas older people’s self-directed activities fostered 2-way communication. Participant A6 remarked that these activities were “chatty and allowed for suggestions,” creating a more active and engaging atmosphere.

Regarding participation motivation, medical staff-led activities benefited from the hospital’s brand effect, with participant A9 citing “trust due to the hospital’s authority.” On the other hand, older people’s self-organized activities enhanced participants’ sense of responsibility and achievement, as noted by A9, who expressed pride in their role as a group leader. In evaluating effectiveness, medical staff-led activities were praised for their “authoritative knowledge and ability to solve practical problems,” rated highly by participants A7 and A10. Meanwhile, older people’s self-directed activities were particularly valued for their “sense of being needed” (A2) and “autonomy” (A9), which fostered sustained participation and reflected participants’ initiative and sense of belonging.

##### Positive Aging Attitudes

Positive aging attitudes gradually appeared in the improvement of self-efficacy and the change of health concepts: A8 demonstrated health autonomy in postoperative self-management, A3 embodied the mentality of “doing something for the older people” by helping others; A9 served as a group leader, and A6 became a volunteer, transforming from a passive receiver to an active contributor and completing the change of social roles. A8 mentioned that “after the doctor’s guidance, I learnt about medication by myself and no longer relied on my children,” and A9 said that “after leading the group to do health exercises, I feel that I still have a role to play.” In terms of health concepts, A3, A6, and A7 shifted from “cure” to “prevention” and paid more attention to daily health care, such as diet and exercise; A10 and A8 strengthened their perception of “health is wealth” due to health crisis events, further increasing their awareness of “health is wealth.”

##### Suggestions and Demands for Improvement

Suggestions and demands for improvement focus on content optimization, activity continuity, resource integration, and so forth. A5, A6, and A7 propose the need to deepen the understanding of highly prevalent diseases, adverse drug reactions, and multidisciplinary consultation, respectively; A6 and A8 propose the use of “short videos to popularize science,” emphasizing the importance of brevity and incorporation of examples, while A3 hopes to add “practical courses,” such as demonstrations of Chinese medicine techniques. A6 and A8 suggested the use of “short videos for popularization,” emphasizing briefness and incorporation of examples, while A3 hoped to add “practical courses,” such as demonstration of Chinese medicine techniques. Regarding activity continuity, A2 suggests once a week, A3 prefers once a month, and A6 emphasizes maintaining a stable frequency. Regarding dissemination channels, A8 relied on children’s notifications, A7 preferred public number push, and a balance between offline and digital means was needed. In integrating resources, A7 and A9 suggested that hospitals regularly send doctors to provide professional guidance, and A3 and A6 hoped that neighborhood committees would strengthen the promotion of activities and expand community coverage. These suggestions reflect the diverse needs of participants regarding health education content, format, and support.

## Discussion

### Principal Findings

The study found that the community health association significantly improved older people’s Health Promotion Lifestyle Scale total score, Self-rated Abilities for Health Practices Scale total score, and healthy self-help behavior total score through a combination of professional guidance and peer interaction (*P*<.001). Qualitative interviews identified that peer demonstration (eg, A6 observing Baduanjin exercises to form exercise habits) and health crisis events (eg, A10 adjusting lifestyle habits after discovering thyroid nodules) jointly drove behavioral change, corroborating the validity of social cognitive theory [31] and low-cost community-based interventions [32]. Compared with traditional 1-way education, our model enhances the behavioral stickiness of older people through the mechanism of “2-way empowerment,” that is, medical staff provide chronic disease management courses to ensure scientific validity, and peer leaders lower the threshold of participation through emotional connection [33-36], forming a sustainable “participation-empowerment” cycle. Notably, cultural relevance significantly influenced intervention effectiveness, with participants showing greater receptiveness to culturally congruent health knowledge, particularly traditional Chinese health care practices.

Our study found that the community health association significantly increased the level of autonomous participation of older people (*P*=.004), particularly improving social role functioning and indoor autonomy. Health crisis events (eg, self-management after A8 cardiac surgery) triggered self-efficacy awakening, which prompted older people to change from “dependents” to “autonomous managers” [31], a finding consistent with the “role enrichment” effect of social role theory [37]. This transition from passive participation to leadership roles strengthened social connectedness and sense of belonging, aligning with the World Health Organization’s active aging framework emphasizing “health, participation and security” for active aging [38]. The study also found that older people’s health communication ability was improved, such as A3 pairing up to help spread health knowledge and A7 integrating online information for family discussion, forming the dual role of “beneficiary-communicator.” This active aging practice provides a sustainable solution for an aging society by activating the willingness to participate through low-cost activities [32]. Our study also observed that older women demonstrated significantly higher participation and engagement levels than men. In China, older women typically assume greater household health management responsibility. They are more accustomed to participating in community activities, increasing their willingness to engage in health intervention programs [39]. Conversely, older male participants may be more inclined to view seeking health assistance as a sign of weakness, which to some extent constitutes a barrier to their participation in community health activities [39,40].

Our study constructed a hybrid model of “online lightweight learning—offline immersive interaction,” significantly improving older people’s eHEALS scores (*P*=.002). Interviews showed that older people improved their functional skills (A6: learning Baduanjin through short videos), critical skills (A7: actively identifying false information on public websites), and interactive applications (A9: forming online health communities). In particular, A9 spontaneously formed an online health community after mastering the function of WeChat forwarding health information through the intervention course, reflecting the transformation from technical learning to social participation [41]. This result validates the eHealth Literacy Framework’s theoretical pathway of “skills training—contextual practice—confidence enhancement” [42]. Compared with previous intervention studies, the online activities of community health associations provide older people with readily accessible learning resources and flexible learning modes. Offline activities focus on establishing interpersonal interactions and facilitating knowledge translation and application, lowering the participation threshold for technologically disadvantaged groups, and effectively resolving the difficulties of older people in applying technology [43].

### Policy Implications and Action Recommendations

To ensure the sustainable development of this intervention model, we propose the following recommendations. First, it is recommended that the community health association model be integrated into the routine operational framework of primary public health services, with its functional role clearly defined and stable collaboration established with community health service centers. Second, standard operating procedures and training manuals should be developed to facilitate replication in other communities. Utilizing digital platforms can expand coverage at a low cost and reduce expenses. Finally, we should explore diverse funding sources, including expanding support from public welfare funds and social enterprises. A certification and incentive system for community senior volunteers should be established to encourage social participation among older people while ensuring the stability of the volunteer workforce.

### Limitations

Our study has the following limitations: the study included a majority of female subjects and was concentrated in urban areas, which limits the representativeness of the sample and may limit the universality of the conclusions to be adopted. In the future, the sample size needs to be expanded to verify the model’s applicability in a pluralistic social context. The measurement tools used in the study were all self-assessment scales, which may have the risk of social desirability bias, response bias, and other factors. In future research, objective physiological indicators (such as body measurements and electronic health records) can be incorporated into the evaluation system to validate the impact of intervention programs on real-world health outcomes. Our study used its own pre-post controlled experimental design, which could not entirely exclude the interference of time effects on the results, despite the low mobility and relatively stable health status of older people in the community. Due to this reason, our study revealed the deeper causes of behavioral change through qualitative interviews and compensated for the limitations of a single design with a mixed methods approach. In the future, a stepped hybrid design can be further adapted to promote multicenter studies and gradually increase the strength of evidence. In addition, the current qualitative study did not explore the barriers and facilitating factors affecting project implementation. This limitation restricts the research’s generalization and impacts policymakers’ understanding and promotion of the program. Subsequent research will explore the barriers and facilitating factors affecting project implementation in depth, aiming to provide decision-making support for the program’s broader community implementation.

### Conclusion

Our study demonstrates that the CHSE-O has successfully constructed a primary empowerment health promotion program through low-cost, community-based education. The model successfully stimulates healthy self-help behaviors among older adults while enhancing their social participation. By fostering a virtuous cycle of “self-help—mutual help—social contribution,” it potentially provides a replicable community-based solution to improve access to health care resources. Our research findings provide a reference for designing sustainable, participant-led health intervention programs and underscore the value of investing in self-sustaining health programs. Future studies can explore the underlying mechanisms of behavioral change, conduct rigorous cost-benefit analyses, and evaluate this model’s long-term efficacy and cost-effectiveness across diverse sociocultural contexts.

## Supplementary material

10.2196/81062 Multimedia Appendix 1 The implementation and analysis processes of the semistructured interviews.

10.2196/81062 Multimedia Appendix 2 Analysis of the association between changes in primary outcomes (Δ) and demographic characteristics.

10.2196/81062 Multimedia Appendix 3 General information of the interviewees.

## Abbreviations

CHSE-O: community healthy self-help education model for older adults
eHEALS: eHealth Literacy Scale
IPA-I: Impact on Participation and Autonomy
