# Supplementary material for: From Passive to Active—Improving the Healthy Self-Help Behavior of Older Adults Through Community Health Association: Mixed Methods Study
Source: J Med Internet Res. 2025 Nov 25;27:e81062. doi: 10.2196/81062 (PMC12646557; doi:10.2196/81062)
Supplement: Multimedia Appendix 1 [file jmir-v27-e81062-s001.docx]

**Appendix 1: The implementation and analysis processes of the semi-structured interviews**

**Semi-structured Interviews**

Interviewers, who had received standardized training in qualitative research methods, conducted face-to-face interviews with 15-20 experts. The entire process was audio-recorded, and non-verbal information (such as facial expressions and body movements) was recorded simultaneously. The interview outline included the following questions:

(1) In the active health-based community self-help health education model for older adults, what are the design concepts and considerations regarding the course content?

(2) How should the active health-based community self-help health education model for older adults be implemented and constructed? What are the specific implementation methods and future development directions?

The expert panel meeting was held in a quiet and comfortable hospital conference room and lasted for 220 minutes. The interviewer actively listened, appropriately followed up on participants' responses, and carefully recorded non-verbal cues such as facial expressions, body language, and speech rate. The results indicated that experts considered a core educational model to be a "lecture + practice + interaction" composite educational framework, guided by "practicality + fun" and integrating Traditional Chinese Medicine (TCM) theory with modern health management techniques. The research team's discussion led to the following modifications:

**I. Course Content Design Principles and Content Optimization**

1. Regarding the content in the teaching package related to the operation and explanation of digital technology principles, one expert suggested that, considering the digital literacy and comprehension difficulties of older adults, the teaching content and process should be made as easy-to-understand as possible. Additionally, an on-site Q&A session should be arranged after the lecture. The expert panel agreed and updated the teaching package content to enhance the suitability of the courses for the community self-help health education of older adults.

2. One expert proposed teaching based on "integrating seasonal health preservation with TCM theory" to enhance cultural identity. Furthermore, regarding the course pace, an expert pointed out the need to optimize content in real-time based on classroom feedback and control the duration of single lectures. Flexible teaching content and timing should be formed according to the audience type, age, and specific teaching methods.

3. One expert noted that adopting a combined "lecture + practice" model (e.g., Baduanjin teaching + health checks) received support from most experts. Simultaneously, organizing free clinics with doctors from multiple departments would increase the practicality of the courses (e.g., thyroid tumor screening); adding sessions like "liver-protection exercise demonstrations" and "learning experience sharing" would strengthen the subjective initiative of older adults; and for seniors living alone, adding "one-on-one tutoring" and community information support mechanisms. These decisions would ultimately support and improve individual and collective health. The expert panel adopted this suggestion.

**II. Project Implementation Methods and Future Development Directions**

1. Regarding project implementation, experts suggested increasing differentiation by classifying needs according to urban/rural areas and age groups and strengthening the focus accordingly (e.g., focusing on first-aid knowledge in urban communities, and chronic disease management in rural areas). Additionally, parts of the "TCM medication guidance" content were highly accepted and could be listed as mandatory course content in the project arrangement.

2. Based on suggestions from two experts, a cooperation mechanism was incorporated into the original project's education model. The "peer education" model was adopted for the education mode, encouraging mutual help among older adults, and distributing graphic manuals to aid memory. Establishing a "community-hospital" linkage system (e.g., Shuguang Hospital collaborating with residential committees) would ensure resource integration and utilization. Inviting doctors from cross-departments into communities regularly would form a long-term service chain.

3. For sustainability, experts suggested developing layered course modules, such as a basic class (health common sense) and an advanced class (disease management), to disseminate knowledge multi-dimensionally across gradients. They also proposed using multimedia tools (e.g., videos on making health-preserving hammers) to enhance fun and attract sustained participation from older adults. Comprehensive dissemination of knowledge content through new media would form the core project modules: TCM health preservation, digital skills, disease prevention, and community mutual aid.
