# Supplementary material for: From Passive to Active—Improving the Healthy Self-Help Behavior of Older Adults Through Community Health Association: Mixed Methods Study
Source: J Med Internet Res. 2025 Nov 25;27:e81062. doi: 10.2196/81062 (PMC12646557; doi:10.2196/81062)
Supplement: Multimedia Appendix 2 [file jmir-v27-e81062-s002.docx]

**Multimedia Appendix 2:** Analysis of the Association Between Changes in Primary Outcomes (Δ) and Socio-demographic Characteristics (n=80)

| **Variables** |  | **Number** | **Percent（%）** | **ΔHPLP II**  **(**$\overline{\boldsymbol{x}}$***±s*)** | **ΔSRAHP**  **(**$\overline{\boldsymbol{x}}$***±s*)** | **ΔHealthy Self-help Behavior**  **(**$\overline{\boldsymbol{x}}$***±s*)** | **ΔIPA-Ⅰ**  **(**$\overline{\boldsymbol{x}}$***±s*)** | **ΔeHEALS**  **(**$\overline{\boldsymbol{x}}$***±s*)** | ***P* value** |
| --- | --- | --- | --- | --- | --- | --- | --- | --- | --- |
| **Gender** |  |  |  |  |  |  |  |  |  |
|  | Male | 15 | 18.8 | 30.33±17.13 | 34.73±15.34 | 23.67±17.04 | 16.2±8.95 | 8.6±5.8 |  |
|  | Female | 65 | 81.3 | 31.86±23.06 | 32.8±21.76 | 26.34±22.38 | 19.54±15.14 | 9.18±6.52 |  |
|  | *t* |  |  | -0.241 | 0.325 | -0.433 | -0.819 | -0.319 | >0.05 |
| **Age** |  |  |  |  |  |  |  |  |  |
|  | 60~65 | 22 | 26.8 | 30.32±21.74 | 34.73±20.08 | 26.00±19.84 | 13.91±9.13 | 9.73±6.76 |  |
|  | 66~70 | 32 | 39.0 | 29.56±25.01 | 30.34±24.39 | 23.53±24.34 | 23.44±17.54 | 8.91±7.3 |  |
|  | 71~75 | 18 | 22.0 | 32.22±15.94 | 34.17±13.37 | 29.44±16.19 | 19.06±10.73 | 8.33±4.54 |  |
|  | 76~80 | 5 | 6.1 | 38.60±26.69 | 35.40±25.21 | 22.60±28.09 | 12.00±14.02 | 7.00±5.00 |  |
|  | ＞80 | 3 | 3.7 | 46.67±16.92 | 42.00±16.64 | 33.00±26.00 | 18.00±15.00 | 14.00±3.46 |  |
|  | *r* |  |  | 0.138 | 0.056 | 0.049 | 0.086 | -0.015 | >0.05 |
| **Educational level** |  |  |  |  |  |  |  |  |  |
|  | Junior high and below | 48 | 60.0 | 32.00±22.05 | 31.60±19.00 | 25.83±22.41 | 21.25±14.73 | 9.77±6.69 |  |
|  | High school/secondary vocational | 23 | 28.7 | 27.74±18.62 | 33.83±21.17 | 23.35±17.82 | 12.04±9.97 | 7.96±5.63 |  |
|  | College/university | 9 | 11.3 | 39.11±29.20 | 39.78±28.03 | 32.22±25.28 | 24.00±15.65 | 8.22±6.53 |  |
|  | *r* |  |  | -0.023 | 0.071 | 0.049 | -0.148 | -0.143 | >0.05 |
| **Religious beliefs** |  |  |  |  |  |  |  |  |  |
|  | No | 67 | 83.8 | 32.27±22.32 | 34.27±20.32 | 24.96±21.15 | 18.68±14.20 | 8.89±6.58 |  |
|  | Yes | 13 | 16.3 | 28.00±20.56 | 27.46±22.12 | 30.38±22.99 | 20.07±14.8 | 10.00±5.21 |  |
|  | *F* |  |  | 0.408 | 1.188 | 0.698 | 0.103 | 0.325 |  |
| **Marital status** |  |  |  |  |  |  |  |  |  |
|  | Unmarried | 1 | 1.3 | 36.00 | 25.00 | 23.00 | 14.00 | 15.00 |  |
|  | Married | 66 | 82.5 | 32.78±22.65 | 33.98±21.16 | 27.12±21.72 | 19.68±14.74 | 9.15±6.59 |  |
|  | Divorce | 1 | 1.3 | 13.00 | 35.00 | 3.00 | 8.00 | 8.00 |  |
|  | Widow | 12 | 15.0 | 26.08±19.00 | 29.16±19.58 | 20.91±20.46 | 16.00±11.72 | 8.25±5.44 |  |
|  | *F* |  |  | 0.559 | 0.233 | 0.667 | 0.46 | 0.36 | >0.05 |
| **Residential situation** |  |  |  |  |  |  |  |  |  |
|  | Living alone | 13 | 16.3 | 26.31±12.26 | 22.69±11.71 | 15.62±13.26 | 15.31±9.4 | 8.85±4.95 |  |
|  | Living with spouse | 42 | 52.5 | 36.12±22.79 | 38.07±21.57 | 31.62±21.73 | 19.86±13.59 | 9.48±7.25 |  |
|  | Living with children | 10 | 12.5 | 26.8±28.33 | 31.2±25.67 | 25.9±27.76 | 18.4±15.28 | 7.2±4.85 |  |
|  | Living with spouse and children | 15 | 18.8 | 26.6±20.72 | 29.8±17.6 | 18.47±17.35 | 19.73±18.87 | 9.4±5.94 |  |
|  | *F* |  |  | 1.274 | 2.163 | 2.778 | 0.353 | 0.355 | >0.05 |
| **Household monthly income** |  |  |  |  |  |  |  |  |  |
|  | <5000 CNY | 45 | 56.3 | 29.84±22.99 | 29.51±19.68 | 24.93±23.59 | 20.73±15.94 | 9.04±6.85 |  |
|  | 5001-8000 CNY | 16 | 20.0 | 25.56±15.6 | 35.5±21.87 | 21.81±16.83 | 14.94±10.26 | 7.88±3.79 |  |
|  | 8001-10000 CNY | 8 | 10.0 | 42.25±19.58 | 46.75±19.93 | 32.75±21.84 | 14。00±9.18 | 10.75±7.55 |  |
|  | >10000 CNY | 11 | 13.8 | 39.64±24.94 | 34.82±21.07 | 30.36±17.98 | 20.82±13.97 | 9.73±6.84 |  |
|  | *r* |  |  | 0.17 | 0.215 | 0.15 | -0.058 | 0.038 | >0.05 |
| **Primary source of income** |  |  |  |  |  |  |  |  |  |
|  | Pension | 75 | 93.8 | 30.37±21.65 | 32.60±19.90 | 24.99±20.83 | 18.52±13.98 | 9.09±6.45 |  |
|  | Child support | 1 | 1.3 | 55.00 | 73.00 | 72.00 | 27.00 | 8.00 |  |
|  | Re-employment income | 2 | 2.5 | 40.00±24.04 | 17.50±20.51 | 18.5±23.33 | 14.00±4.24 | 5.50±7.78 |  |
|  | Other | 2 | 2.5 | 56.50±28.99 | 50.00±35.36 | 42.00±26.87 | 34.50±28.99 | 12.50±3.54 |  |
|  | *F* |  |  | 1.432 | 2.183 | 2.136 | 1.011 | 0.404 | >0.05 |
| **Medical Payment Methods** |  |  |  |  |  |  |  |  |  |
|  | Urban Medical Insurance | 61 | 76.3 | 30.7±19.31 | 32.97±19.16 | 25.41±19.55 | 17.7±13.9 | 8.93±5.82 |  |
|  | Rural Medical Insurance | 11 | 13.8 | 34.82±31.86 | 30.36±24.84 | 24±29.74 | 22.55±13.55 | 10.64±9.88 |  |
|  | Employee Medical Insurance | 7 | 8.8 | 36.29±29.06 | 42.29±26.84 | 35.71±23.03 | 23.86±18.67 | 8.14±5.05 |  |
|  | Self-financed | 1 | 1.3 | 16.00 | 12.00 | 3.00 | 18.00 | 7.00 |  |
|  | *F* |  |  | 0.377 | 0.872 | 0.908 | 0.663 | 0.308 | >0.05 |
| **Signed ong-term care insurance or not** |  |  |  |  |  |  |  |  |  |
|  | No | 73 | 91.3 | 30.78±20.59 | 33.38±19.99 | 25.40±20.04 | 18.53±13.58 | 9.29±6.12 |  |
|  | Yes | 7 | 8.8 | 39.86±34.41 | 30.86±28.38 | 30.43±34.45 | 22.86±20.56 | 6.86±8.78 |  |
|  | *t* |  |  | -1.044 | 0.308 | -0.592 | -0.767 | 0.965 | >0.05 |
| **Self-assessed health status** |  |  |  |  |  |  |  |  |  |
|  | Very good | 7 | 8.8 | 38.71±32.78 | 41.71±25.23 | 32.71±27.02 | 21.29±17.35 | 10.71±7.83 |  |
|  | Good | 30 | 37.5 | 31.90±24.17 | 34.63±25.54 | 27.03±24.83 | 23.73±16.24 | 10.13±7.45 |  |
|  | Average | 36 | 45.0 | 29.94±19.12 | 31.44±16.10 | 23.61±18.70 | 14.72±11.82 | 8.72±5.23 |  |
|  | Poor | 6 | 7.5 | 33.50±16.36 | 29.17±11.92 | 22.33±12.52 | 16.17±5.23 | 4.17±3.49 |  |
|  | Very poor | 1 | 1.3 | 19.00 | 15.00 | 43.00 | 25.00 | 8.00 |  |
|  | *r* |  |  | -0.088 | -0.17 | -0.087 | -0.201 | -0.212 | >0.05 |
| **Number of chronic diseases** |  |  |  |  |  |  |  |  |  |
|  | 0 | 1 | 1.3 | 75.00 | 78.00 | 71.00 | 36.00 | 8.00 |  |
|  | 1 | 59 | 73.8 | 30.49±22.93 | 32.36±21.82 | 24.15±22.42 | 19.47±15.34 | 9.05±7.02 |  |
|  | 2 | 14 | 17.5 | 30.71±18.81 | 34.29±14.29 | 30.64±17.4 | 17.43±10.89 | 9.57±4.72 |  |
|  | 3 | 6 | 7.5 | 37.00±13.49 | 31.00±15.94 | 23.67±10.42 | 14.00±7.24 | 8.33±2.94 |  |
|  | *r* |  |  | 0.009 | -0.054 | -0.004 | -0.137 | -0.003 | >0.05 |

Note: Δ value = 12-month score - baseline score
