# Supplementary material for: From Passive to Active—Improving the Healthy Self-Help Behavior of Older Adults Through Community Health Association: Mixed Methods Study
Source: J Med Internet Res. 2025 Nov 25;27:e81062. doi: 10.2196/81062 (PMC12646557; doi:10.2196/81062)
Supplement: Multimedia Appendix 3 [file jmir-v27-e81062-s003.docx]

**Multimedia Appendix 3 General information of the interviewees (n=11)**

| Coding | Gender | Age | Educational level | Occupation before retirement | Self-assessed health status | Living habits |
| --- | --- | --- | --- | --- | --- | --- |
| A1 | Female | 69 | High school | Company employee | Good, no chronic diseases | Non-smoker, non-drinker; has hypertension and takes medication regularly; engages in regular physical activity. |
| A2 | Female | 79 | High school | Company employee | Good, no chronic diseases | Non-smoker, non-drinker; has hypertension and diabetes, takes medication regularly; engages in regular physical activity. |
| A3 | Female | 70 | High school | Statistician | Low back pain, no chronic diseases | Non-smoker, non-drinker; engages in regular physical activity; participates in community ‘Old Partners’ activities. |
| A4 | Male | 63 | High school | Taxi driver | Low back pain, no chronic diseases | Non-smoker, non-drinker; maintains regular physical activity and routines; participates in community volunteer services. |
| A5 | Female | 68 | High school | Factory clerk | Lower back pain, higher blood lipids | Engages in regular physical activity; is interested in Traditional Chinese Medicine (TCM) health practices (e.g., moxibustion). |
| A6 | Female | 68 | High school | Factory clerk | High blood lipids, need to take medication | Engages in regular physical activity (Baduanjin); pays attention to Traditional Chinese Medicine (TCM) health practices. |
| A7 | Female | 78 | University | Teacher | hypertension | Engages in regular physical activity; actively seeks health knowledge through channels like Baidu and WeChat public accounts. |
| A8 | Female | 73 | Junior high school | Factory worker | Good control of diabetes, hypertension and hyperlipidaemia after cardiac stenting | Exercises twice daily; takes afternoon naps; maintains early-to-bed, early-to-rise habits; participates in community health promotion activities. |
| A9 | Male | 79 | Junior high school | Farmer | Hypertension, well controlled | Walks and does health exercises daily; follows health information on TV and short video platforms. |
| A10 | Male | 66 | High school | Factory manager | Thyroid nodules, hypertension, and high blood lipids (under medication control) | Walks 6,000-10,000 steps daily; does not engage in other forms of exercise. |
| A11 | Female | 70 | High school | Factory worker | Self-awareness is average, no major diseases | Daily walking of 6,000-7,000 steps; pays attention to iodized salt intake. |
